# NLRX1 Is a Multifaceted and Enigmatic Regulator of Immune System Function

**DOI:** 10.3389/fimmu.2019.02419

**Published:** 2019-10-11

**Authors:** Margaret A. Nagai-Singer, Holly A. Morrison, Irving C. Allen

**Affiliations:** ^1^Department of Biomedical Sciences and Pathobiology, Virginia-Maryland College of Veterinary Medicine, Virginia Tech, Blacksburg, VA, United States; ^2^Department of Basic Science Education, Virginia Tech Carilion School of Medicine, Roanoke, VA, United States

**Keywords:** NOD-like receptor, pattern recognition receptor, TRAFasome, autophagy, interferon, NF-κB, mitochondria

## Abstract

Over the last decade, significant progress has been achieved in defining mechanisms underlying NLR regulation of immune system function. However, several NLR family members continue to defy our best attempts at characterization and routinely exhibit confounding data. This is particularly true for NLR family members that regulate signaling associated with the activation of other pattern recognition receptors. NLRX1 is a member of this NLR sub-group and acts as an enigmatic regulator of immune system function. NLRX1 has been shown to negatively regulate type-I interferon, attenuate pro-inflammatory NF-κB signaling, promote reactive oxygen species production, and modulate autophagy, cell death, and proliferation. However, the mechanism/s associated with NLRX1 modulation of these pathways is not fully understood and there are inconsistencies within the field. Likewise, it is highly likely that the full repertoire of biological functions impacted by NLRX1 are yet to be defined. Recent mouse studies have shown that NLRX1 significantly impacts a multitude of diseases, including cancer, virus infection, osteoarthritis, traumatic brain injury, and inflammatory bowel disease. Thus, it is essential that the underlying mechanism associated with NLRX1 function in each of these diseases be robustly defined. Here, we summarize the current progress in understanding mechanisms associated with NLRX1 function. We also offer insight into both unique and overlapping mechanisms regulated by NLRX1 that likely contribute to disease pathobiology. Ultimately, we believe that an improved understanding of NLRX1 will result in better defined mechanisms associated with immune system attenuation and the resolution of inflammation in a myriad of diseases.

## NLRX1: The Enigmatic NLR

**Figure 1:**
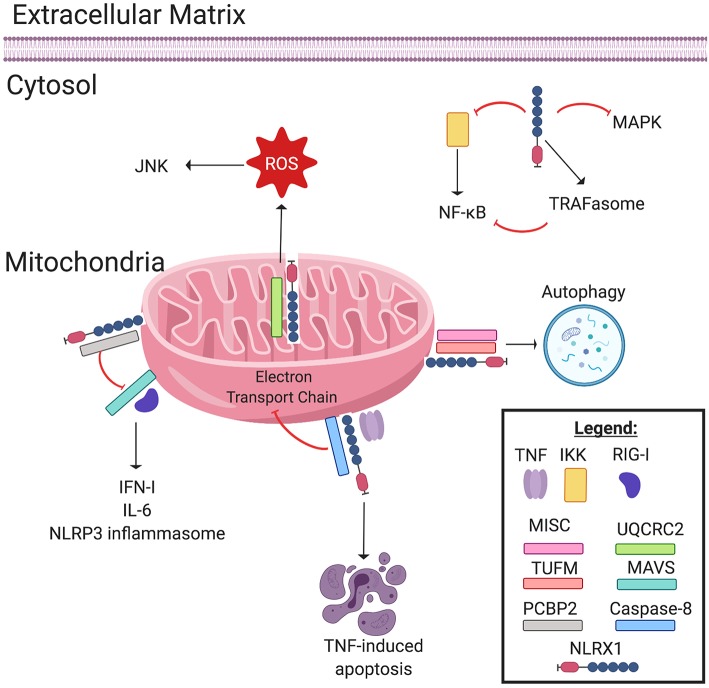
NLRX1 regulates immune system signaling. The Nod-like receptor NLRX1 has many diverse, multifaceted roles in innate immune system signaling, and cellular localization plays a key role in determining NLRX1's function. Localized on and within the mitochondria, NLRX1 interacts with a multitude of pathways. NLRX1 interacts with the complex III associated protein UQCRC2 to promote the production of reactive oxygen species (ROS). ROS in turn activates the JNK pathway, which promotes apoptosis. NLRX1 attenuates MAVS signaling through disruption of RIG-I activation via interactions with poly(rC) binding protein 2 (PCBP2). This negatively regulates the production of IL-6, IFN-1, and possibly NLRP3 inflammasome formation. When associated with the mitochondrial immune signaling complex (MISC) and TUFM, NLRX1 promotes autophagy. Lastly, in the presence of TNF, NLRX1 interacts with Caspase-8 to induce TNF-induced apoptosis, and this interaction may inhibit Complex I and III of the Electron Transport Chain. In the cytosol, NLRX1 inhibits NF-κB signaling by interacting with IκB kinase (IKK). Likewise, cytosolic NLRX1 may promote TRAFasome formation, which in turn inhibits NF-κB signaling. Lastly, NLRX1 may also inhibit the MAPK pathway.

Since the initial description of the NLR family of pattern recognition receptors over 20 years ago, significant progress has been made in understanding their biology. However, NLRX1 remains an enigma. NLRX1 (NOD5/NOD9/CLR11.3) has several atypical features that contribute to its complexity and uniqueness within the NLR family. For example, members of the NLR family are defined by their tripartite domain structure, which includes a variable combination of a limited repertoire of protein domains (typically pyrin or CARD domains) on the N-terminus, a conserved nucleotide binding domain in the central region, and a variable number of leucine rich repeats (LLR) on the C-terminus (1). NLRX1 lacks a fully characterized N-terminus, hence the “X” nomenclature used to define the gene/protein. To date, the only defined domain of the N-terminus of NLRX1 is a mitochondria-targeting sequence (MTS) (2–5). The C-terminus of NLRX1 is also unique, consisting of 7 LRRs followed by an uncharacterized three-helix bundle (6). This three-helix bundle likely has a range of diverse functions, potentially including participation in molecular recognition and scaffolding. NLRX1 is considered to be ubiquitously expressed in mammalian cells, with evidence supporting cell type specific differences in function (2, 7, 8). Like the other NLR family members, NLRX1 appears to function as a scaffolding protein following activation and facilitates the formation of multiprotein complexes. However, the full range of pathogen-associated- and damage-associated molecular patterns sensed by NLRX1 is far from clear and the interacting proteins are only minimally characterized. The current dogma in the NLR field places NLRX1 in a unique sub-family of regulatory NLRs that are non-inflammasome forming and function, in part, through the regulation of inflammation signaling associated with the activation of other pattern recognition receptors (9). Other NLRs in this sub-family include NOD1, NOD2, NLRC3, and NLRP12 (9). NOD1 and NOD2 are positive regulatory NLRs, as they augment inflammatory signaling networks. NLRX1, NLRC3, and NLRP12 function as negative regulatory NLRs, thought to attenuate overzealous immune system activation and likely participate in inflammation resolution (9). Specifically, NLRX1 has been shown to negatively regulate NF-κB and type-I interferon (IFN-I) signaling, modulate the production of reactive oxygen species (ROS), participate in autophagy and cell death, and impact JNK and MAPK pathways (Figure 1). This review will explore the proposed mechanisms by which NLRX1 affects these processes and attempt to provide insight into this mysterious NLR family member.

## NLRX1 Attenuates Pattern Recognition Receptor Signaling in the Cytosol

The majority of well-characterized NLRs function as cytosolic sensors, where upon activation, they act as a scaffold to form multiprotein complexes and promote inflammation. NLRX1 has also been found in the cytoplasm (10, 11). However, as mentioned above and similar to NLRC3 and NLRP12, cytosolic NLRX1 functions as a negative regulator of inflammation (12). While all three of these negative regulatory NLRs likely have other functions in the cytosol, all appear to attenuate inflammation through targeting components of the NF-κB signaling pathway (12). In the case of NLRX1, activation results in an interaction with TRAF6 (7, 10). At baseline, NF-κB are bound to the inhibitor IκB and NLRX1 appears to be complexed with TRAF6 in the cytosol (10). Following activation, IκB Kinase (IKK) phosphorylates IκB, resulting in its degradation and freeing the NF-κB for nuclear transit and transcription initiation (10). However, in the presence of lipopolysaccharide likely associated with TLR4 activation, NLRX1 and TRAF6 undergo K63-linked polyubiquitination resulting in complex disassociation (10). Once detached, the LRR domain of NLRX1 binds to the kinase domain of the activated IKK complex, resulting in the attenuation of the NF-κB pathway (10). The targeting of TRAFs is not unique to NLRX1. Both NLRC3 and NLRP12 have been shown to interact with TRAF6 and TRAF3, resulting in the respective attenuation of either canonical or non-canonical NF-κB signaling pathways (7, 13). The multiprotein complex that forms between these specific NLRs and the respective TRAF family members has been dubbed the “TRAFasome” (12, 13). However, significant mechanistic details pertaining to the signals leading up to TRAFasome formation, the temporal regulation of the pathways, additional biological functions regulated by the multiprotein complex, and other proteins directly or indirectly involved in complex formation are not fully understood. It should be noted that NLRX1 attenuation of NF-κB signaling has been predominately defined in the context of host-pathogen interactions. However, several studies have also characterized this mechanism underlying NLRX1's role as a tumor suppressor in various types of cancer (Table 1) (8, 28).

**Table 1:** NLRX1 modulates diverse diseases and host-pathogen interactions.

**Disease/infection**	**Mechanism**	**References**
Acute myocardial ischemia (AMI)	NLRX1 has a protective role in myocardial ischemic injury by inhibiting inflammation and hypoxia-induced apoptosis.	(14)
Breast cancer	NLRX1 modulates mitochondrial functions to suppress tumorigenesis in solid tumors, but may facilitate aggressive breast cancer metastasis.	(15)
*Chlamydia trachomatis*	ROS production induced by NLRX1 creates optimal conditions for Chlamydial growth.	(16)
Chronic Obstructive Pulmonary Disease (COPD)	NLRX1 expression is suppressed in murine models of CS-induced activation of the inflammasome and lungs of human COPD patients. NLRX1 likely inhibits CS-induced pulmonary inflammation by regulating MAVS.	(17, 18)
Colitis-associated cancer/Sporadic Colon Cancer	In *Nlrx1^−/−^* CAC murine models, mice were more susceptible to CAC pathogenesis. Increased signaling of common cancer-promoting pathways including NF-κB, MAPK, STAT3, and IL-6 were observed.	(8)
Colitis/Inflammatory Bowel Disease (IBD)	NLRX1 has a protective role against IBD due to its effect on the microbiome and negative regulation of inflammation.	(19)
Deafness (aging and neomycin induced)	NLRX1 aggravates apoptosis of cochlear hairs and may play a role in hair cell maturity.	(20)
Experimental Autoimmune Encephalomyelitis (EAE)/Multiple Sclerosis (MS)	NLRX1 is protective against neurological diseases by negatively regulating inflammation. NLRX1 may be protective against additional neurological diseases, including Parkinson's and Alzeihmer's diseases, by maintaining glutamate homeostasis in the central nervous system.	(21–23)
Hepatitis C (HCV)	NLRX1 promotes HCV infection by interacting with PCBP2 to inhibit MAVS via K48-linked polyubiquitination.	(24)
*Helicobacter pylori*	*Helicobacter pylori* infection promotes inflammation and can lead to gastric cancer. NLRX1 expression is decreased in Chinese gastric cancer patients.	(25)
Hepatocellular carcinoma (HCC)	NLRX1 expression is decreased in human HCC patients.	(26, 27)
Histiocytic sarcoma	NLRX1 may suppress tumorigenesis by inhibiting NF-κB signaling in mice.	(28)
Hyperglycemia	Decreased expression of NLRX1 may be protective against diet-induced hyperglycemia due to decreased pancreatic lipid accumulation.	(29)
Human immunodeficiency virus (HIV)	NLRX1 expression affects HIV infections, but seems to act controversially. NLRX1 expression is decreased in human HIV patients, but NLRX1 promotes establishment of latent HIV-1 reservoirs in mice.	(30–32)
Influenza A Virus (IAV)	NLRX1 interacts with the influenza PB1-F2 protein to protect macrophages from apoptosis, but also downregulates IFN-β and IL-6 production.	(7, 33)
Kaposi's sarcoma-associated herpesvirus (KSHV)	NLRX1 decreases IFN-1 production, which encourages KSHV to reactivate out of the latency stage.	(34)
*Listeria monocytogenes*	NLRX1 promotes *L. monocytogenes*- induced mitophagy, helping *L. monocytogenes* to evade killing.	(35)
Nonalcoholic steatohepatitis (NASH)	NLRX1 expression is decreased in NASH mouse models.	(36)
Osteoarthritis (OA)	NLRX1 has a protective role in OA. When upregulated, NLRX1 inhibits NF-κB signaling, which inhibits LPS-induced apoptosis and inflammation in chondrocytes that contribute to OA.	(37)
Periodontitis	NLRX1 expression is increased in human adult periodontitis patient samples.	(38)
Porcine Reproductive and Respiratory Syndrome Virus (PRRSV)	NLRX1 interacts with Nsp9 to restrict viral replication.	(39)
Preterm birth	NLRX1 is expressed in human placenta, amnion, and choriodecidua samples, suggesting that it may play a role in preterm birth-related inflammation.	(40)
Renal ischemia-reperfusion injury	NLRX1 is protective in mouse models of renal ischemia-reperfusion injury, and NLRX1 expression is reduced in human kidney samples with ischemic injury.	(41)
Rheumatoid arthritis (RA)	NLRX1 expression is significantly decreased in human RA patient synovial tissue samples.	(42)
Rhinovirus	NLRX1 interaction with Rhinovirus RNA promotes ROS production, leading to the disruption of epithelial barrier function in the airway.	(43)
*Salmonella enterica* serovar Enteritidis (SE)	NLRX1 is significantly upregulated in the follicles of ducks that are susceptible to SE and SE-infected ducks. NLRX1 is believed to increase recognition of SE by the host.	(44)
*Shigella flexneri*	NLRX1 promotes ROS production activated by *Shigella* infection, which promotes signaling pathways dependent on NF-κB and Jun amino-terminal kinases (JNK).	(3)
Systemic Lupus Erythematosus (SLE)	NLRX1 expression did not affect MAVS aggregation, but cytosolic NLRX1 was found in SLE patients.	(11)
Traumatic brain injury (TBI)	*Nlrx1^−/−^* murine models have significantly increased NF-κB signaling, which contributes to increased numbers of microglia and macrophages in cortical lesions. NLRX1 is significantly decreased in human post-aneurysm brain injury patients.	(45)
Type 2 Diabetes Mellitus (T2DM)/Diabetic Nephropathy (DM)	NLRX1 polymorphism rs4245191 is a risk factor for T2DM complications including macrovascular complications and cerebral infarction due to its mutated form. Interestingly, NLRX1 does not have a role in DN.	(46, 47)

In addition to negatively regulating NF-κB signaling, an intriguing hypothesis has also been proposed that suggests NLRX1 actually shuttles from the cytosol to the mitochondria to regulate inflammation and mitochondrial functions (12). Under this postulated scenario, once released from TRAF6 as described above, NLRX1 transits alone or in complex with a currently unidentified chaperone/s to the mitochondria. Consistent with this hypothesis, several other NLRs shuttle between cellular compartments. For example, NLRC5 and CIITA/NLRA can translocate from the cytosol to the nucleus to regulate inflammation signaling during virus infection (12, 48–51). Similarly, NOD1 and NOD2 have also been shown to shuttle between the cytosol and the plasma membrane (12, 52). As NLRX1 lacks many of the traditional translocation sequences, the mechanism underlying how NLRX1 may move between cellular compartments is still unclear. However, its ability to form multiprotein complexes opens the possibility of interactions with potential chaperones. For example, several NLRs have been shown to interact with Heat Shock Proteins, which are critical molecular chaperones for driving translocation between cellular compartments (53–57). Consistent with this hypothesis, HSP90 has been shown to interact with NLRP12 and controls its negative regulation of non-canonical NF-κB signaling (53). NLRX1 has been localized in the cytoplasm on different layers of the mitochondria, and even in mitochondrial granules (2, 4, 5, 7, 11, 24, 58). Each of these locations have significant biological implications that potentially impact NLRX1 function.

## NLRX1 Regulates Immune System Function through Mitochondria Localization

In addition to negatively regulating NF-κB signaling, NLRX1 has also been shown to directly modulate pattern recognition receptor signaling associated with Rig-I-like Helicase Receptors (RLRs) (2). Specifically, NLRX1 inhibits the interaction between two RLRs, RIG-I and MDA5, and the Mitochondrial Anti-Viral Signaling (MAVS) protein following virus exposure to attenuate IFN-I signaling (2, 7, 10, 59, 60). MAVS is an adaptor protein located on the outer mitochondrial membrane. It is used by RIG-I to restrict virus infection by activating NF-κB and IFN regulatory factor 3 and 7 (IRF3 and IRF7) to produce IL-6 and IFN-I (2, 10, 24). Additionally, it is necessary for MAVS-dependent NLRP3 inflammasome formation (14). Mechanistically, NLRX1 was originally shown to form a multiprotein complex with MAVS on the outer membrane of the mitochondria and compete with RIG-I/MDA5 binding to MAVS (2, 12). This original model suggests the C-terminal LRR of NLRX1 is responsible for preventing MAVS from producing IFNs (2). This mechanism has been somewhat refined in more recent studies. It is now postulated that in the presence of viral RNA, the nucleotide-binding domain of NLRX1 interacts with MAVS and poly(rC) binding protein 2, causing K48-linked polyubiquitination of MAVS (24). This degradation inhibits MAVS, leading to a suppressed immune response due to decreased IFN production and inflammation. Regardless of which domain is responsible for interacting with MAVS, decreased IFN levels put the host at a higher risk for infections like HIV, HCV, influenza, and Kaposi's sarcoma-associated herpesvirus reactivation (7, 24, 30, 34). However, attenuation of inflammation is also critical to maintain immune system homeostasis during the process of resolution once the pathogen has been cleared and also protects the host from autoimmune disorders (61). Indeed, dysfunctional NLRX1 has been associated with several autoimmune diseases including lupus, multiple sclerosis, and inflammatory bowel disease (Table 1) and is expressed in a multitude of cell and tissue types associated with these maladies (2, 7, 11, 19, 21, 22, 62).

Many of the mechanisms ascribed to NLRX1 and multiprotein complex formation have been based on other better characterized NLRs. For example, other NLRs have also been shown to form multiprotein complexes with MAVS to regulate IFN signaling following virus infection (12). Following either RSV or VSV exposure, NOD2 interacts with MAVS and this interaction is required for proper IFN signaling in both hematopoietic and non-hematopoietic cells (63). However, consistent with their positive and negative regulatory functions, the NOD2-MAVS interaction exacerbates IFN signaling and inflammation; whereas, the NLRX1-MAVS interaction attenuates these processes (2, 12). The regulation of MAVS is complex, as other molecules like PSMA7, FAF1, STING, PB1-F2, and PKR might function concurrently with NLRX1 to impact innate immunity. A subunit of the proteasome PSMA7 functions similarly to NLRX1, decreasing IFN-I production by inhibiting MAVS (64). Likewise, NLRX1 further hinders IFN-I production by binding to STING, a component of MAVS signaling, to disrupt the STING-TBK1 interaction (30, 65). On the other hand, FAF1 disrupts the NLRX1-MAVS complex, freeing MAVS to activate pro-inflammatory pathways and produce IFN-I (66). NLRX1 also competes with PKR to initiate an antiviral response by protecting IRF1 function (67). This mechanism appears to be specific as NLRX1 prevents IRF3 expression to inhibit MAVS, but allows IRF1 activation (67). Contrastingly, some believe that NLRX1 does not associate with MAVS, but rather interacts directly with viral proteins, like PB1-F2 on the influenza A virus (33). It is possible, and even likely, that other unidentified proteins interact with NLRX1 to negatively regulate inflammation and anti-viral host responses. The complexity of this regulation contributes to the confounding data seen related to NLRX1 and MAVS. Indeed, there are many aspects of these mitochondrial mechanisms that are still undefined, including the temporal dynamics of the interactions, other proteins that may participate either directly or indirectly in potential NLRX1 multi-protein complex formation, and cell or microbial signals necessary to trigger either positive or negative regulation.

In addition to its role in modulating MAVS signaling on the outer membrane of the mitochondria, NLRX1 has also been shown to be localized within the mitochondria on the inner membrane and matrix (3, 4). Internalized NLRX1 interacts with the protein UQCRC2 in the electron transport chain (4). This interaction has been suggested to potentiate the production of ROS from the mitochondria (4). NLRX1 mediated modulation of ROS production by the mitochondria has significant implications in multiple biological functions, including anti-viral immunity and cancer. ROS production results in the activation of multiple transcription factors, including NF-κB, and is a potent damage associated molecular pattern that is sensed by several pattern recognition receptors, such as NLRP3 (68, 69). Increased oxidative stress is also a key driver of cell death through JNK signaling activation and a significant contributing factor in tumorigenesis, cisplatin-induced ototoxicity, and bacterial infections (3, 16, 70–73). Thus, while the negative regulatory effects of NLRX1 on inflammation are well-documented, this unique NLR also acts to augment ROS production that can promote inflammation. While this may seem counterintuitive, it is likely that the biological impact of the increased ROS production is to facilitate apoptosis, which is a typical host-defense mechanism following virus infection and during tumorigenesis, rather than drive inflammation. Specifically, studies have suggested NLRX1 does so by activating JNK signaling through the production of ROS, and interactions with Caspase-8 (3, 72, 74). For example, NLRX1 has been shown to be required for rhinovirus-mediated disruptions to the airway epithelial barrier (43). In this study, NLRX1 silencing resulted in the elimination of both virus-associated and poly(I:C)-associated ROS production and was shown to be essential for rhinovirus induced NOX-1 expression in polarized airway epithelial cells (43). This attenuation of NLRX1 and subsequent elimination in mitochondrial ROS production was associated with improved cell survival, tight junction formation, and barrier function (43). Contrastingly, NLRX1 reportedly exerts protective effects against apoptosis in chondrocytes and tubular epithelial cells, and the modulation of apoptosis may be dependent on its interactions with yet another protein, SARM1 (37, 41, 75).

## NLRX1 Regulates Multiple Biological Functions Through the Modulation of Autophagy

Beyond the diverse roles discussed thus far, NLRX1 has also been shown to modulate autophagy. Autophagy is a critical biological process associated with cell death, inflammation, and tumorigenesis. In the context of viral pathogenesis, autophagy upregulation is associated with improved virus clearance. Intracytoplasmic virions can be captured within the autophagy pathway and transferred to lysosomes for eventual breakdown and/or pattern recognition receptor sensing, resulting in the activation of innate and adaptive immune responses (76). NLRX1's promotion and regulation of autophagy has been reported in several instances within the context of virus exposure (59, 60). These studies reveal that NLRX1 is capable of augmenting autophagy pathways by associating with the TUFM protein (59). TUFM is a molecule that not only potently suppresses RIG-I signaling, but is also associated with the autophagy complex ATG12–ATG5–ATG16L1. NLRX1 and TUFM appear to act together to keep IFN-I production in check and also prevent decreases in autophagy (59, 60). The ATG12–ATG5 complex can also interact directly with MAVS to inhibit IFN-I. For example, its absence has been shown to lead to accumulation of MAVS on the mitochondria and elevation of IFN-I (60). Thus, while NLRX1 seems to enhance autophagy, this may actually augment its negative regulation of IFN-I.

In addition to interactions with TUFM during virus infection, NLRX1 has also been shown to modulate autophagy though interactions with the Beclin 1-UVRAG complex. This complex is critical for regulating autophagy following bacteria exposure (77). In studies with Group A Streptococcus, cell invasion was significantly increased in the absence of NLRX1 (77). This was associated with a decrease in autophagosome and autolysome formation (77). Mechanistically, NLRX1 was shown to interact with Beclin 1 through its NACHT domain and function as a negative regulator to inactivate the Beclin 1-UVRAG complex following bacteria invasion (77). Presumably, the negative regulation of this inhibitory complex actually enhances the binding capacity of Beclin 1 with additional proteins, such as Atg14L. This shift from a Beclin 1-UVRAG complex to a Beclin 1-Atg14L complex is predicted to promote autophagy and increase endolysosomal trafficking (78).

Furthermore, intriguing data has recently revealed that NLRX1 also plays a role in mitophagy in the context of both infectious disease and cancer (15, 35). Mitophagy is a process cells use to purge damaged or unnecessary mitochondria. Pathogens often exploit this mechanism to evade host recognition and killing. For example, the virulence factor listeriolysin O from *L. monocytogenes* induces mitophagy in macrophages (35). NLRX1 was shown to promote *L. monocytogenes*-induced mitophagy (35). NLRX1 is the only NLR family member with a MTS that contains an LC3-interacting region that directly associates with LC3 (35). This oligomerization was induced by listeriolysin O, resulting in mitophagy (35). Conversely, NLRX1 deficiency was found to increase mitochondrial production of ROS and reduced bacteria survival (35). Additionally, the interaction with LC3 modulates proinflammatory cytokine production by macrophages in response to fungal infection (79). In the context of cancer, NLRX1 plays a role in TNF induced mitochondria-lysosomal crosstalk in mammary tumors (15). NLRX1 appears to maintain the crosstalk between mitochondrial metabolism and lysosomal function to modulate key cancer hallmarks (15). When NLRX1 is deleted, lysosomal function is impaired and turnover of damaged mitochondria through mitophagy is reduced (15). This results in decreased OxPhos-dependent cell proliferation and breast cancer cell migration ability in the presence of TNF (15). Together, these studies show the importance of NLRX1 in mitophagy and further identify it as a potential target for future therapeutic interventions.

## Conclusions

There is significantly more to the NLR family beyond the formation of the inflammasome. Over the last two decades, our understanding of the regulatory NLR family members that function to either augment or attenuate signaling associated with other families of pattern recognition receptors has greatly increased our overall understanding of immune system regulation. The recent characterization of NLRs that function as negative regulators, which participate in the attenuation of inflammation and promote resolution underscore the point that many NLR family members have yet to be significantly characterized. Even among NLRs that have been relatively well-studied, including NLRX1, conflicting data in the literature is common. However, there is a general consensus regarding the broad mechanisms associated with this unique NLR, including regulation of NF-κB, IFN-I signaling, autophagy, and ROS production. However, more mechanistic insight is certainly needed to better define the high-resolution details of its role in each of these biological processes and signaling pathways. As NLRX1 potentially contributes to a multitude of human diseases (Table 1), it is critical to better characterize this enigmatic NLR to propel the field forward and bolster the development of novel disease treatments.

## Author Contributions

MN-S, HM, and IA conducted literature reviews, analyzed and interpreted data, prepared the figures, and wrote the manuscript. IA provided content expertise and overall direction. All authors have read and approved the manuscript.

### Conflict of Interest

The authors declare that the research was conducted in the absence of any commercial or financial relationships that could be construed as a potential conflict of interest.

## References

[1] Schroder K Tschopp J. The inflammasomes. Cell. (2010) 140:821–32. 10.1016/j.cell.2010.01.040 20303873

[2] Moore CB Bergstralh DT Duncan JA Lei Y Morrison TE Zimmermann AG. NLRX1 is a regulator of mitochondrial antiviral immunity. Nature. (2008) 451:573–7. 10.1038/nature06501 18200010

[3] Tattoli I Carneiro LA Jehanno M Magalhaes JG Shu Y Philpott DJ. NLRX1 is a mitochondrial NOD-like receptor that amplifies NF-kappaB and JNK pathways by inducing reactive oxygen species production. EMBO Rep. (2008) 9:293–300. 10.1038/sj.embor.7401161 18219313 PMC2267388

[4] Arnoult D Soares F Tattoli I Castanier C Philpott DJ Girardin SE. An N-terminal addressing sequence targets NLRX1 to the mitochondrial matrix. J Cell Sci. (2009) 122(Pt 17):3161–8. 10.1242/jcs.051193 19692591 PMC2871076

[5] Song X Li W Xie X Zou Z Wei J Wu H. NLRX1 of black carp suppresses MAVS-mediated antiviral signaling through its NACHT domain. Dev Comp Immunol. (2019) 96:68–77. 10.1016/j.dci.2019.03.001 30853538

[6] Reubold TF Hahne G Wohlgemuth S Eschenburg S. Crystal structure of the leucine-rich repeat domain of the NOD-like receptor NLRP1: implications for binding of muramyl dipeptide. FEBS Lett. (2014) 588:3327–32. 10.1016/j.febslet.2014.07.017 25064844

[7] Allen IC Moore CB Schneider M Lei Y Davis BK Scull MA. NLRX1 protein attenuates inflammatory responses to infection by interfering with the RIG-I-MAVS and TRAF6-NF-kappaB signaling pathways. Immunity. (2011) 34:854–65. 10.1016/j.immuni.2011.03.026 21703540 PMC3166771

[8] Koblansky AA Truax AD Liu R Montgomery SA Ding S Wilson JE. The innate immune receptor NLRX1 functions as a tumor suppressor by reducing colon tumorigenesis and key tumor-promoting signals. Cell Rep. (2016) 14:2562–75. 10.1016/j.celrep.2016.02.064 26971998 PMC4853907

[9] Allen IC. Non-inflammasome forming NLRs in inflammation and tumorigenesis. Front Immunol. (2014) 5:169. 10.3389/fimmu.2014.00169 24795716 PMC4001041

[10] Xia X Cui J Wang HY Zhu L Matsueda S Wang Q. NLRX1 negatively regulates TLR-induced NF-kappaB signaling by targeting TRAF6 and IKK. Immunity. (2011) 34:843–53. 10.1016/j.immuni.2011.02.022 21703539 PMC3150212

[11] Shao WH Shu DH Zhen Y Hilliard B Priest SO Cesaroni M. Prion-like aggregation of mitochondrial antiviral signaling protein in lupus patients is associated with increased levels of type I interferon. Arthritis Rheumatol. (2016) 68:2697–707. 10.1002/art.39733 27110677 PMC5079845

[12] Coutermarsh-Ott S Eden K Allen IC. Beyond the inflammasome: regulatory NOD-like receptor modulation of the host immune response following virus exposure. J Gen Virol. (2016) 97:825–38. 10.1099/jgv.0.000401 26763980 PMC4854363

[13] Schneider M Zimmermann AG Roberts RA Zhang L Swanson KV Wen H. The innate immune sensor NLRC3 attenuates Toll-like receptor signaling via modification of the signaling adaptor TRAF6 and transcription factor NF-kappaB. Nat Immunol. (2012) 13:823–31. 10.1038/ni.2378 22863753 PMC3721195

[14] Li H Zhang S Li F Qin L. NLRX1 attenuates apoptosis and inflammatory responses in myocardial ischemia by inhibiting MAVS-dependent NLRP3 inflammasome activation. Mol Immunol. (2016) 76:90–7. 10.1016/j.molimm.2016.06.013 27393910

[15] Singh K Roy M Prajapati P Lipatova A Sripada L Gohel D. NLRX1 regulates TNF-alpha-induced mitochondria-lysosomal crosstalk to maintain the invasive and metastatic potential of breast cancer cells. Biochim Biophys Acta Mol Basis Dis. (2019) 1865:1460–76. 10.1016/j.bbadis.2019.02.018 30802640

[16] Abdul-Sater AA Said-Sadier N Lam VM Singh B Pettengill MA Soares F. Enhancement of reactive oxygen species production and chlamydial infection by the mitochondrial Nod-like family member NLRX1. J Biol Chem. (2010) 285:41637–45. 10.1074/jbc.M110.137885 20959452 PMC3009891

[17] Kang MJ Yoon CM Kim BH Lee CM Zhou Y Sauler M. Suppression of NLRX1 in chronic obstructive pulmonary disease. J Clin Invest. (2015) 125:2458–62. 10.1172/JCI71747 25938787 PMC4497738

[18] Kang MJ Shadel GS. A mitochondrial perspective of chronic obstructive pulmonary disease pathogenesis. Tuberc Respir Dis. (2016) 79:207–13. 10.4046/trd.2016.79.4.207 27790272 PMC5077724

[19] Leber A Hontecillas R Tubau-Juni N Zoccoli-Rodriguez V Abedi V Bassaganya-Riera J. NLRX1 modulates immunometabolic mechanisms controlling the host-gut microbiota interactions during inflammatory bowel disease. Front Immunol. (2018) 9:363. 10.3389/fimmu.2018.00363 29535731 PMC5834749

[20] Yang Q Sun G Cao Z Yin H Qi Q Wang J. The expression of NLRX1 in C57BL/6 mice cochlear hair cells: possible relation to aging- and neomycin-induced deafness. Neurosci Lett. (2016) 616:138–46. 10.1016/j.neulet.2015.11.053 26836140

[21] Eitas TK Chou WC Wen H Gris D Robbins GR Brickey J. The nucleotide-binding leucine-rich repeat (NLR) family member NLRX1 mediates protection against experimental autoimmune encephalomyelitis and represses macrophage/microglia-induced inflammation. J Biol Chem. (2014) 289:4173–9. 10.1074/jbc.M113.533034 24366868 PMC3924282

[22] Gharagozloo M Gris KV Mahvelati T Amrani A Lukens JR Gris D. NLR-dependent regulation of inflammation in multiple sclerosis. Front Immunol. (2017) 8:2012. 10.3389/fimmu.2017.02012 29403486 PMC5778124

[23] Mahmoud S Gharagozloo M Simard C Amrani A Gris D. NLRX1 enhances glutamate uptake and inhibits glutamate release by astrocytes. Cells. (2019) 8:E400. 10.3390/cells8050400 31052241 PMC6562695

[24] Qin Y Xue B Liu C Wang X Tian R Xie Q. NLRX1 mediates MAVS degradation to attenuate hepatitis C virus-induced innate immune response through PCBP2. J Virol. (2017) 91:e01264–17. 10.1128/JVI.01264-17 28956771 PMC5686720

[25] Castano-Rodriguez N Kaakoush NO Goh KL Fock KM Mitchell HM. The NOD-like receptor signalling pathway in *Helicobacter pylori* infection and related gastric cancer: a case-control study and gene expression analyses. PLoS ONE. (2014) 9:e98899. 10.1371/journal.pone.0098899 24901306 PMC4047072

[26] Wang X Yang C Liao X Han C Yu T Huang K. NLRC and NLRX gene family mRNA expression and prognostic value in hepatocellular carcinoma. Cancer Med. (2017) 6:2660–72. 10.1002/cam4.1202 28960882 PMC5673949

[27] Hu B Ding GY Fu PY Zhu XD Ji Y Shi GM. NOD-like receptor X1 functions as a tumor suppressor by inhibiting epithelial-mesenchymal transition and inducing aging in hepatocellular carcinoma cells. J Hematol Oncol. (2018) 11:28. 10.1186/s13045-018-0573-9 29482578 PMC5828065

[28] Coutermarsh-Ott S Simmons A Capria V LeRoith T Wilson JE Heid B. NLRX1 suppresses tumorigenesis and attenuates histiocytic sarcoma through the negative regulation of NF-kappaB signaling. Oncotarget. (2016) 7:33096–110. 10.18632/oncotarget.8861 27105514 PMC5078078

[29] Costford SR Tattoli I Duan FT Volchuk A Klip A Philpott DJ. Male mice lacking NLRX1 are partially protected from high-fat diet-induced hyperglycemia. J Endocr Soc. (2018) 2:336–47. 10.1210/js.2017-00360 29577109 PMC5855099

[30] Mar KB Schoggins JW. NLRX1 helps HIV avoid a STING operation. Cell Host Microbe. (2016) 19:430–1. 10.1016/j.chom.2016.03.011 27078064

[31] Nasi M De Biasi S Bianchini E Digaetano M Pinti M Gibellini L. Analysis of inflammasomes and antiviral sensing components reveals decreased expression of NLRX1 in HIV-positive patients assuming efficient antiretroviral therapy. AIDS. (2015) 29:1937–41. 10.1097/QAD.0000000000000830 26237098

[32] Barouch DH Ghneim K Bosche WJ Li Y Berkemeier B Hull M. Rapid inflammasome activation following mucosal SIV infection of rhesus monkeys. Cell. (2016) 165:656–67. 10.1016/j.cell.2016.03.021 27085913 PMC4842119

[33] Jaworska J Coulombe F Downey J Tzelepis F Shalaby K Tattoli I. NLRX1 prevents mitochondrial induced apoptosis and enhances macrophage antiviral immunity by interacting with influenza virus PB1-F2 protein. Proc Natl Acad Sci USA. (2014) 111:E2110–9. 10.1073/pnas.1322118111 24799673 PMC4034189

[34] Ma Z Hopcraft SE Yang F Petrucelli A Guo H Ting JP. NLRX1 negatively modulates type I IFN to facilitate KSHV reactivation from latency. PLoS Pathog. (2017) 13:e1006350. 10.1371/journal.ppat.1006350 28459883 PMC5426799

[35] Zhang Y Yao Y Qiu X Wang G Hu Z Chen S. Listeria hijacks host mitophagy through a novel mitophagy receptor to evade killing. Nat Immunol. (2019) 20:433–46. 10.1038/s41590-019-0324-2 30804553

[36] Wang YG Fang WL Wei J Wang T Wang N Ma JL. The involvement of NLRX1 and NLRP3 in the development of nonalcoholic steatohepatitis in mice. J Chin Med Assoc. (2013) 76:686–92. 10.1016/j.jcma.2013.08.010 24084392

[37] Ma D Zhao Y She J Zhu Y Zhao Y Liu L. NLRX1 alleviates lipopolysaccharide-induced apoptosis and inflammation in chondrocytes by suppressing the activation of NF-kappaB signaling. Int Immunopharmacol. (2019) 71:7–13. 10.1016/j.intimp.2019.03.001 30861394

[38] Ebersole JL Kirakodu S Novak MJ Exposto CR Stromberg AJ Shen S. Effects of aging in the expression of NOD-like receptors and inflammasome-related genes in oral mucosa. Mol Oral Microbiol. (2016) 31:18–32. 10.1111/omi.12121 26197995 PMC4712099

[39] Jing H Song T Cao S Sun Y Wang J Dong W. Nucleotide-binding oligomerization domain-like receptor X1 restricts porcine reproductive and respiratory syndrome virus-2 replication by interacting with viral Nsp9. Virus Res. (2019) 268:18–26. 10.1016/j.virusres.2019.05.011 31132368 PMC7114581

[40] Bryant AH Bevan RJ Spencer-Harty S Scott LM Jones RH Thornton CA. Expression and function of NOD-like receptors by human term gestation-associated tissues. Placenta. (2017) 58:25–32. 10.1016/j.placenta.2017.07.017 28962692

[41] Stokman G Kors L Bakker PJ Rampanelli E Claessen N Teske GJD. NLRX1 dampens oxidative stress and apoptosis in tissue injury via control of mitochondrial activity. J Exp Med. (2017) 214:2405–20. 10.1084/jem.20161031 28626071 PMC5551566

[42] Kim HW Kwon YJ Park BW Song JJ Park YB Park MC. Differential expressions of NOD-like receptors and their associations with inflammatory responses in rheumatoid arthritis. Clin Exp Rheumatol. (2017) 35:630–7. 28240593

[43] Unger BL Ganesan S Comstock AT Faris AN Hershenson MB Sajjan US. Nod-like receptor X-1 is required for rhinovirus-induced barrier dysfunction in airway epithelial cells. J Virol. (2014) 88:3705–18. 10.1128/JVI.03039-13 24429360 PMC3993547

[44] Zhang Y Chen Y Gu T Xu Q Zhu G Chen G. Effects of *Salmonella enterica* serovar Enteritidis infection on egg production and the immune response of the laying duck Anas platyrhynchos. PeerJ. (2019) 7:e6359. 10.7717/peerj.6359 30701142 PMC6348949

[45] Theus MH Brickler T Meza AL Coutermarsh-Ott S Hazy A Gris D. Loss of NLRX1 exacerbates neural tissue damage and NF-kappaB signaling following brain injury. J Immunol. (2017) 199:3547–58. 10.4049/jimmunol.1700251 28993512 PMC5683102

[46] Zeng C Zhou Z Han Y Wen Z Guo C Huang S. Interactions of TRAF6 and NLRX1 gene polymorphisms with environmental factors on the susceptibility of type 2 diabetes mellitus vascular complications in a southern Han Chinese population. J Diabetes Complicat. (2017) 31:1652–7. 10.1016/j.jdiacomp.2017.08.013 29046236

[47] Scantlebery AML Uil M Butter LM Poelman R Claessen N Girardin SE NLRX1 does not play a role in diabetes nor the development of diabetic nephropathy induced by multiple low doses of streptozotocin. PLoS ONE. (2019) 14:e0214437 10.1371/journal.pone.0214437 30908533 PMC6433286

[48] Benko S Magalhaes JG Philpott DJ Girardin SE. NLRC5 limits the activation of inflammatory pathways. J Immunol. (2010) 185:1681. 10.4049/jimmunol.0903900 20610642

[49] Meissner TB Li A Biswas A Lee KH Liu YJ Bayir E. NLR family member NLRC5 is a transcriptional regulator of MHC class I genes. Proc Natl Acad Sci USA. (2010) 107:13794–9. 10.1073/pnas.1008684107 20639463 PMC2922274

[50] Orlandi C Forlani G Tosi G Accolla RS. Molecular and cellular correlates of the CIITA-mediated inhibition of HTLV-2 Tax-2 transactivator function resulting in loss of viral replication. J Transl Med. (2011) 9:106. 10.1186/1479-5876-9-106 21736733 PMC3141499

[51] Tosi G Forlani G Andresen V Turci M Bertazzoni U Franchini G. Major histocompatibility complex class II transactivator CIITA is a viral restriction factor that targets human T-cell lymphotropic virus type 1 Tax-1 function and inhibits viral replication. J Virol. (2011) 85:10719–29. 10.1128/JVI.00813-11 21813598 PMC3187506

[52] Kufer TA Kremmer E Adam AC Philpott DJ Sansonetti PJ. The pattern-recognition molecule Nod1 is localized at the plasma membrane at sites of bacterial interaction. Cell Microbiol. (2008) 10:477–86. 10.1111/j.1462-5822.2007.01062.x 17970764

[53] Arthur JC Lich JD Aziz RK Kotb M Ting JP. Heat shock protein 90 associates with monarch-1 and regulates its ability to promote degradation of NF-kappaB-inducing kinase. J Immunol. (2007) 179:6291–6. 10.4049/jimmunol.179.9.6291 17947705

[54] Yue S Zhu J Zhang M Li C Zhou X Zhou M. The myeloid heat shock transcription factor 1/beta-catenin axis regulates NLR family, pyrin domain-containing 3 inflammasome activation in mouse liver ischemia/reperfusion injury. Hepatology. (2016) 64:1683–98. 10.1002/hep.28739 27474884 PMC5074868

[55] Gutierrez-Lopez TY Orduna-Castillo LB Hernandez-Vasquez MN Vazquez-Prado J Reyes-Cruz G. Calcium sensing receptor activates the NLRP3 inflammasome via a chaperone-assisted degradative pathway involving Hsp70 and LC3-II. Biochem Biophys Res Commun. (2018) 505:1121–7. 10.1016/j.bbrc.2018.10.028 30316511

[56] Swaroop S Mahadevan A Shankar SK Adlakha YK Basu A HSP60 critically regulates endogenous IL-1beta production in activated microglia by stimulating NLRP3 inflammasome pathway. J Neuroinflamm. (2018) 15:177 10.1186/s12974-018-1355-6 PMC5994257 29885667

[57] Wang Y Sedlacek AL Pawaria S Xu H Scott MJ Binder RJ. Cutting Edge: the heat shock protein gp96 activates inflammasome-signaling platforms in APCs. J Immunol. (2018) 201:2209–14. 10.4049/jimmunol.1800505 30209191 PMC6176107

[58] Singh K Sripada L Lipatova A Roy M Prajapati P Gohel D. NLRX1 resides in mitochondrial RNA granules and regulates mitochondrial RNA processing and bioenergetic adaptation. Biochim Biophys Acta Mol Cell Res. (2018) 1865:1260–76. 10.1016/j.bbamcr.2018.06.008 29932989

[59] Lei Y Wen H Yu Y Taxman DJ Zhang L Widman DG. The mitochondrial proteins NLRX1 and TUFM form a complex that regulates type I interferon and autophagy. Immunity. (2012) 36:933–46. 10.1016/j.immuni.2012.03.025 22749352 PMC3397828

[60] Lei Y Wen H Ting JP. The NLR protein, NLRX1, and its partner, TUFM, reduce type I interferon, and enhance autophagy. Autophagy. (2013) 9:432–3. 10.4161/auto.23026 23321557 PMC3590269

[61] Fekete T Bencze D Szabo A Csoma E Biro T Bacsi A. Regulatory NLRs control the RLR-mediated type I interferon and inflammatory responses in human dendritic cells. Front Immunol. (2018) 9:2314. 10.3389/fimmu.2018.02314 30344524 PMC6182093

[62] Leber A Hontecillas R Tubau-Juni N Zoccoli-Rodriguez V Hulver M McMillan R. NLRX1 regulates effector and metabolic functions of CD4(+) T cells. J Immunol. (2017) 198:2260–8. 10.4049/jimmunol.1601547 28159898 PMC5340590

[63] Sabbah A Chang TH Harnack R Frohlich V Tominaga K Dube PH. Activation of innate immune antiviral responses by Nod2. Nat Immunol. (2009) 10:1073–80. 10.1038/ni.1782 19701189 PMC2752345

[64] Jia Y Song T Wei C Ni C Zheng Z Xu Q. Negative regulation of MAVS-mediated innate immune response by PSMA7. J Immunol. (2009) 183:4241–8. 10.4049/jimmunol.0901646 19734229

[65] Guo H Konig R Deng M Riess M Mo J Zhang L. NLRX1 sequesters STING to negatively regulate the interferon response, thereby facilitating the replication of HIV-1 and DNA viruses. Cell Host Microbe. (2016) 19:515–28. 10.1016/j.chom.2016.03.001 27078069 PMC4833116

[66] Kim JH Park ME Nikapitiya C Kim TH Uddin MB Lee HC. FAS-associated factor-1 positively regulates type I interferon response to RNA virus infection by targeting NLRX1. PLoS Pathog. (2017) 13:e1006398. 10.1371/journal.ppat.1006398 28542569 PMC5456407

[67] Feng H Lenarcic EM Yamane D Wauthier E Mo J Guo H. NLRX1 promotes immediate IRF1-directed antiviral responses by limiting dsRNA-activated translational inhibition mediated by PKR. Nat Immunol. (2017) 18:1299–309. 10.1038/ni.3853 28967880 PMC5690873

[68] Gloire G Legrand-Poels S Piette J. NF-kappaB activation by reactive oxygen species: fifteen years later. Biochem Pharmacol. (2006) 72:1493–505. 10.1016/j.bcp.2006.04.011 16723122

[69] Allen IC Scull MA Moore CB Holl EK McElvania-TeKippe E Taxman DJ. The NLRP3 inflammasome mediates *in vivo* innate immunity to influenza A virus through recognition of viral RNA. Immunity. (2009) 30:556–65. 10.1016/j.immuni.2009.02.005 19362020 PMC2803103

[70] Klaunig JE Xu Y Isenberg JS Bachowski S Kolaja KL Jiang J. The role of oxidative stress in chemical carcinogenesis. Environ Health Perspect. (1998) 106:289–95. 10.1289/ehp.98106s1289 9539021 PMC1533298

[71] Circu ML Aw TY. Reactive oxygen species, cellular redox systems, and apoptosis. Free Radic Biol Med. (2010) 48:749–62. 10.1016/j.freeradbiomed.2009.12.022 20045723 PMC2823977

[72] Yin H Sun G Yang Q Chen C Qi Q Wang H. NLRX1 accelerates cisplatin-induced ototoxity in HEI-OC1 cells via promoting generation of ROS and activation of JNK signaling pathway. Sci Rep. (2017) 7:44311. 10.1038/srep44311 28287190 PMC5347132

[73] Yin H Yang Q Cao Z Li H Yu Z Zhang G. Activation of NLRX1-mediated autophagy accelerates the ototoxic potential of cisplatin in auditory cells. Toxicol Appl Pharmacol. (2018) 343:16–28. 10.1016/j.taap.2018.02.007 29454061

[74] Singh K Poteryakhina A Zheltukhin A Bhatelia K Prajapati P Sripada L. NLRX1 acts as tumor suppressor by regulating TNF-alpha induced apoptosis and metabolism in cancer cells. Biochim Biophys Acta. (2015) 1853:1073–86. 10.1016/j.bbamcr.2015.01.016 25639646

[75] Killackey SA Rahman MA Soares F Zhang AB Abdel-Nour M Philpott DJ. The mitochondrial Nod-like receptor NLRX1 modifies apoptosis through SARM1. Mol Cell Biochem. (2019) 453:187–96. 10.1007/s11010-018-3444-3 30191480

[76] Shoji-Kawata S Levine B. Autophagy, antiviral immunity, and viral countermeasures. Biochim Biophys Acta. (2009) 1793:1478–84. 10.1016/j.bbamcr.2009.02.008 19264100 PMC2739265

[77] Aikawa C Nakajima S Karimine M Nozawa T Minowa-Nozawa A Toh H. NLRX1 negatively regulates group A streptococcus invasion and autophagy induction by interacting with the Beclin 1-UVRAG complex. Front Cell Infect Microbiol. (2018) 8:403. 10.3389/fcimb.2018.00403 30488027 PMC6246980

[78] Wu S He Y Qiu X Yang W Liu W Li X. Targeting the potent Beclin 1-UVRAG coiled-coil interaction with designed peptides enhances autophagy and endolysosomal trafficking. Proc Natl Acad Sci USA. (2018) 115:E5669–78. 10.1073/pnas.1721173115 29866835 PMC6016778

[79] Huang JH Liu CY Wu SY Chen WY Chang TH Kan HW. NLRX1 facilitates histoplasma capsulatum-induced LC3-associated phagocytosis for cytokine production in macrophages. Front Immunol. (2018) 9:2761. 10.3389/fimmu.2018.02761 30559741 PMC6286976

